# Value of contrast-enhanced ultrasonography in radiofrequency ablation of secondary hyperparathyroidism

**DOI:** 10.1080/0886022X.2021.1889601

**Published:** 2021-03-04

**Authors:** Xiachuan Qin, Baofu Wang, Boliang Li, Changwei Lin, Xuebin Liu, Xisheng Xie

**Affiliations:** a Department of Ultrasound, Nanchong Central Hospital, The Second Clinical Medical College, North Sichuan Medical College (University), Nan Chong, Peoples Republic of China; b Department of Nephrology, Nanchong Central Hospital, The Second Clinical Medical College, North Sichuan Medical College (University), Nan Chong, Peoples Republic of China

**Keywords:** Hyperplastic parathyroid gland, radiofrequency ablation, contrast-enhanced ultrasound, secondary hyperparathyroidism, chronic kidney disease

## Abstract

**Objectives:**

The purpose of the current study was to determine the performance of contrast-enhanced ultrasound (CEUS) in the assessment of radiofrequency ablation (RFA) of hyperplastic parathyroid glands due to secondary hyperparathyroidism (SHPT).

**Methods:**

Thirty-two patients, each with ≥4 hyperplastic parathyroid glands due to SHPT, underwent RFA *via* hydro-dissection. CEUS was performed in each patient before and during RFA. The patients in whom the intact parathyroid hormone (iPTH) level did not decrease to 300 pg/ml were examined by CEUS. The iPTH, serum calcium, and serum phosphorus levels before and after RFA were compared.

**Results:**

Ablation was achieved in all patients (131 ablated glands). The volume of the glands was 479.88 ± 549.3mm^3^. The pre-operative and day 1 post-operative iPTH levels were 2355 ± 1062 and 292.7 ± 306.8 pg/ml, respectively. Three nodules in three patients showed little enhancement on CEUS on post-operative day 1. The iPTH level was <300 pg/mL on post-operative day 1 in 23 patients, which indicated complete ablation; follow-up evaluations were therefore performed. The pre- and post-operative iPTH levels in the 23 patients were 2113 ± 787.2 and 106.2 ± 84.62 pg/ml, respectively (*p* < 0.05), and the 6- and 12-month post-operative iPTH levels were 111.1 ± 56.57 and 117.6 ± 97.08 pg/ml, respectively (*p* > 0.05).

**Conclusions:**

CEUS-guided RFA is effective and feasible for the treatment of ≥4 hyperplastic parathyroid glands. CEUS was shown to assist the surgeon before, during, and after RFA. CEUS on post-operative day 2, but not immediately post-operatively, was shown to accurately reflect gland perfusion.

## Introduction

Secondary hyperparathyroidism (SHPT) is a common complication of end-stage renal disease. The main manifestations are parathyroid hyperplasia and an abnormal increase in intact parathyroid hormone (iPTH) secretion [1,2]. The incidence of SHPT in hemodialysis patients exceeds 32% [3]. Calcium and phosphorus metabolic disorders are common [4] and the risk of cardiovascular death is increased [3,4]. The long-term survival and quality of life of patients with SHPT are severely affected. Parathyroidectomy (PTX) is the preferred treatment for severe drug-resistant SHPT [5]; however, some patients are unable or unwilling to undergo traditional surgery due to severe cardiopulmonary dysfunction, inability to tolerate general anesthesia, and repeated surgical neck procedures. Radiofrequency ablation (RFA) is widely used for the treatment of various organs [6–9] and RFA has gradually become another treatment method for SHPT patients after surgery [8]. RFA has the following advantages: minimally invasive; performed under local anesthesia; easy to perform; repeated implementation; and short-term efficacy [6–9].

High-quality RFA can completely eliminate lesions while avoiding the loss of adjacent normal tissues [10]. Compared with ultrasound, contrast-enhanced ultrasound (CEUS) greatly improves spatial resolution, accurately assesses the tumor microvessels, and differentiates the tumor from surrounding tissues [11]. CEUS facilitates the locate the position of the lesions pre-operatively, avoids intra-operative risk, and evaluates the curative effect post-operatively.Although CEUS has been widely used in ablation of the liver, kidneys, and thyroid [7,10,12,13], the application of CEUS in SHPT RFA has not been reported. The purpose of this prospective study was to determine the performance of CEUS as an adjunct to RFA in the treatment of hyperplastic parathyroid glands among patients with SHPT.

## Materials and methods

This preliminary and feasibility study was approved by the Institutional Review Board of Nanchong Central Hospital (No：2020008). Written informed consent was obtained from each subject before RFA and CEUS.

### Patients

**Figure 1.**
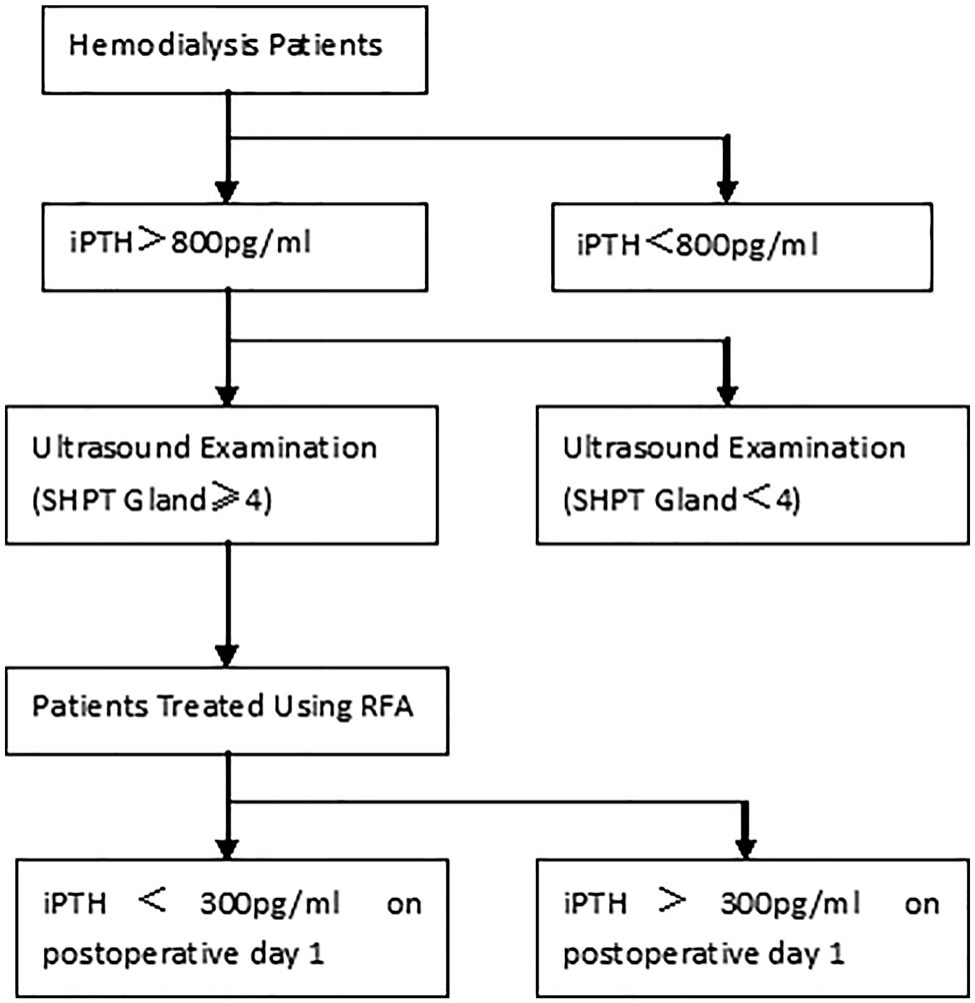
Screening process of inclusion criteria for radiofrequency ablation(RFA) of hyperplastic parathyroid glands due to secondary hyperparathyroidism (SHPT).

A total of 32 consecutive patients with SHPT were subjected to ultrasound-guided RFA between November 2018 and May 2019. The inclusion criteria were as follows: (1) a history of renal insufficiency complicated by SHPT, resistance to drug therapy, no history of SHPT surgery, or other interventional therapy; (2) pre-ablation iPTH level >800 pg/ml; (3) ultrasound examination showing ≥4 hyperplastic SHPT nodules [8]; and (4) nuclide scanning performed to exclude an ectopic parathyroid gland in the mediastinum. The exclusion criteria were as follows: (1) injury to the recurrent laryngeal nerve; (2) declined surgery (3) severe anemia, hemorrhage, or abnormal coagulation profile which contraindicated general anesthesia. The screening process for inclusion of SHPT patients with RFA is shown in Figure 1.

### CEUS examination

A color Doppler ultrasound system (EPIQ 7 C; Philips Ultrasound, Bothell, WA, USA) with a high-frequency linear probe (L12-3) was used to monitor and guide the ablation procedure with a second-generation contrast agent (SonoVue; Bracco, Milan, Italy). CEUS was performed after a bolus injection of SonoVue (1.2 mL) using a mechanical index (MI)<0.1, followed by a 5-mL saline flush. Before RFA treatment, the number, shape, size, and location of nodules were determined by routine ultrasound scanning. CEUS was used to describe the blood supply to the lesion before and after ablation.

The size and position of the gland were observed by injecting contrast medium again after the liquid isolation zone was established. After ablation of one gland, CEUS was performed again. A lack of enhancement in or around the lesion after RFA treatment indicated that the lesion has been completely inactivated. If there is enhancement within or on the edge of the lesion, indicating a residual lesion, supplemental RFA treatment was performed for the residual lesion. The decrease in the iPTH level on the 2^nd^day post-RFA treatment determined whether CEUS was performed. If the iPTH was> 300 pg/ml the next day, CEUS was performed again to assess the presence of a residual lesion.

### RFA technique

RFA of the hyperplastic parathyroid glands was performed using a radiofrequency generator (Taishanglide, Mianyang, China) with an 18-gauge monopolar, internally cooled electrode (Taishanglide). The radiofrequency electrode shaft length was 7 cm with a 0.7 cm active tip. The radiofrequency electrode was cooled by a water circulation pump. Before ablation, CEUS was used to confirm the parathyroid gland blood supply and the ablation route was also determined. Routine disinfection and draping were performed. Under ultrasound guidance, 1% lidocaine was injected to achieve superficial nerve block anesthesia of the neck. Saline was injected around the parathyroid gland under ultrasound guidance, and a separation zone with a thickness of at least 5mmwas established to separate the gland from the surrounding tissues. The ablation needle was punctured into the parathyroid gland, and the ablation was carried out by gradually withdrawing the needle from the lower pole of the posterior margin of the hyperplastic gland. The total ablation was superimposed on the ablation area to cover all of the glands [9]. One side of the parathyroid gland was ablated intra-operatively and no symptoms related to recurrent laryngeal nerve injury were noted. Then, the other parathyroid gland was ablated.

### Clinicopathologic variables

The following data were collected for each patient: nodule volume calculated according to the formula (V = (a × b × c) × Π/6); number of nodules; total parathyroid volume per patient; pre-operative iPTH, serum calcium, and serum inorganic phosphorus levels; and post-operative day 1 iPTH, serum calcium, and serum inorganic phosphorus levels. Calcium was administered orally or intravenously after RFA.An iPTH level <300 pg/mL on post-operative day 1 indicated successful RFA [14]. The serum iPTH level was determined 6 and 12 months after ablation.

### Statistical analysis

All statistical analyses were performed using SPSS software (version 19.0; Chicago, IL, USA). Baseline data are expressed as the mean ± standard deviation (SD). A paired-samples t-test was used to compare variables with a normal distribution between the two groups with respect to serum calcium and serum inorganic phosphorus levels. A *p* value < 0.05 was considered significant.

## Results

### Patient characteristics

A total of 131 parathyroid glands were ablated in 32 patients; there were five glands in three patients and four glands in 29 patients. There were 13 men and 19 women (53.53 ± 13.64 years of age). All patients reported significant improvement in bone pain and/or pruritus within 2 days after ablation (100%).

The post-operative iPTH and serum calcium levels were decreased. The pre- and post-operative day 1iPTH levels were 2355 ± 1062 and 292.7 ± 306.8 pg/ml, respectively (*p* < 0.05). The pre- and post-operative day 1 serum calcium levels were 2.36 ± 0.22 and 1.78 ± 0.28 mmol/l, respectively (*p* < 0.05). The pre- and post-operative day 1 serum inorganic phosphorus levels were 2.06 ± 0.55 and 1.48 ± 0.43 mmol/l, respectively (*p* < 0.05).

### CEUS before RFA

There were 131 glands normally positioned glands. The volume of the glands was 479.88 ± 549.3mm^3^ (range, 25–3218 mm^3^). The total parathyroid volume was 1902.81 ± 1274.7mm^3^ (range, 603–5609 mm^3^). There was a linear correlation between the total parathyroid volume and pre-operative iPTH levels (Pearson correlation coefficient = 0.565, *p* < 0.05). The larger parathyroid volume, the higher the iPTH level. Ninety glands were oval and round-like, 41 of the glands were lobulated, and 1 gland was comma-shaped. All nodules were hypoechoic.

The pre-operative CEUS examination showed rapid hyper-enhancement in the arterial phase in 83 parathyroid glands among 20 patients. Therefore, the perfusion velocity in the nodules was earlier than the perfusion velocity in the thyroid gland. Thirty-six glands among nine patients had equal enhancement compared with the surrounding thyroid gland. Three patients had rapid enhancement on one side and iso-enhancement on the contralateral side.

### CEUS during RFA

During the ablation, one patient had bleeding due to severe vomiting. CEUS showed that the injury was from the right inferior thyroid artery. Under CEUS guidance, ablation was performed and hemostasis was achieved. After cauterization, CEUS showed the formation of a pseudoaneurysm (18 × 11 × 29 mm^3^ in size). Thirty-six hours after the procedure, the pseudoaneurysm was filled with thrombus.

The sound changed after ablation of one gland and the ablation was terminated early in two patients. One patient was unable to endure ablation termination because of severe intra-operative pain. The remaining patient successfully completed the ablation.

### CEUS after RFA

CEUS was carried out immediately post-operatively in patients who successfully completed the operation. None of the115 glands in 28 patients showed enhancement, which was consistent with complete ablation.

Nine of 32 patients had hoarseness (28%); 2 of the 9 patients coughed while drinking water, thus suggesting recurrent laryngeal nerve injuries. Two patients recovered within 1 week, three patients recovered within 3 months, and four patients recovered 6 months after ablation.

**Figure 2.**
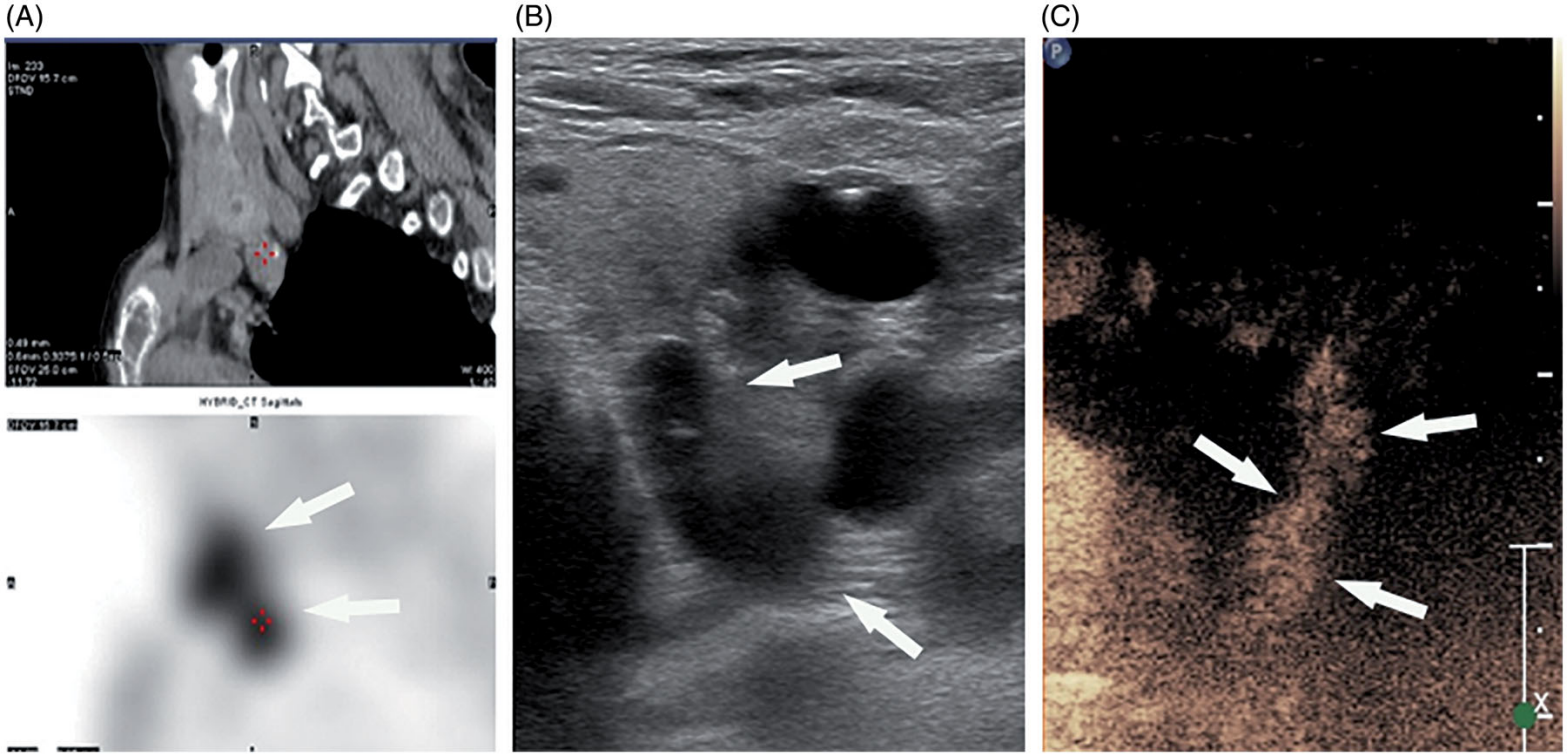
(A) 99mTc-MIBI shows that the nodule has concentration of radioactivity (arrow) in the late phase of the 99mTc-sestamibi sequence. (B) Axial US image shows SHPT hypoechoic nodule boundary behind the lateral lobe of the thyroid and between the carotid artery and trachea before RFA (arrow). (C) After the ablation, contrast-enhanced ultrasonography (CEUS) showed little enhancement of the glands near the trachea (arrow).

**Figure 3.**
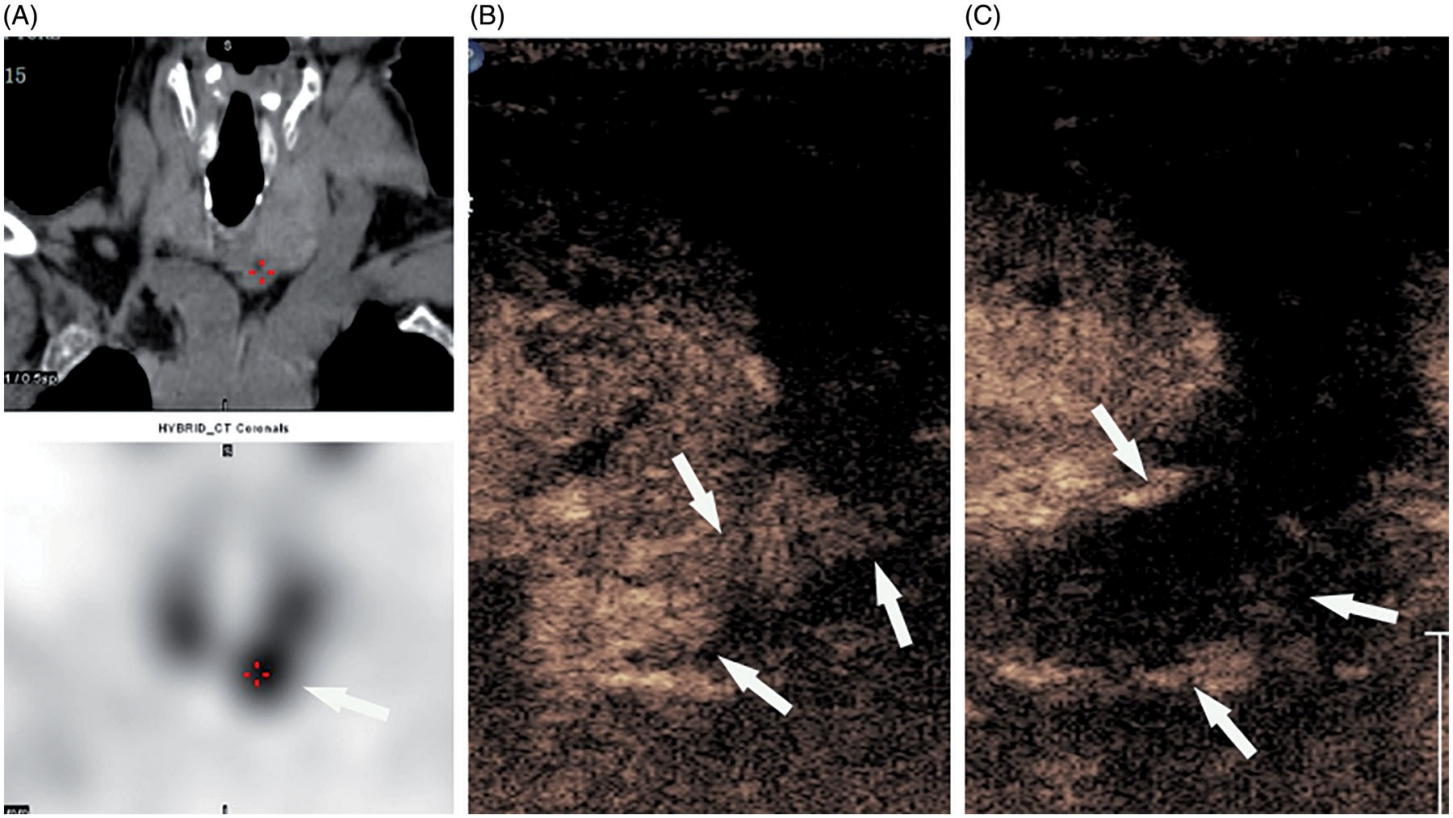
(A) 99mTc-MIBI shows that the nodule has concentration of radioactivity (arrow) in the late phase of the 99mTc-sestamibi sequence. (B) After the first ablation, CEUS revealed a "tail-shape” (arrow) at the upper margin of the gland. (C) After the second ablation, there was no enhancement in the gland (arrow).

One day post-operatively, the iPTH level did not decrease to 300 pg/ml in five patients, and a CEUS examination was performed to evaluate the possibility of an incomplete ablation.Four nodules in four patients showed little enhancement on CEUS, thus indicating incomplete ablation (Figure 2). One of the four patients had a "tail-shape” over the left lower gland. The heteromorphic parathyroid gland resulted in a pre-operative misdiagnosis and incomplete ablation (Figure 3). CEUS showed no enhancement in all four glands of one patient. Thus, a second nuclide examination was performed, but no abnormality was identified; we speculated that there was a fifth gland which was not detected.

### Changes in biochemical indices after successful RFA

Of 23 patients in whom CEUS indicated complete ablation of the nodules, an iPTH level <300 pg/mL on post-operative day 1. The post-operative iPTH and serum calcium levels decreased. The pre- and post-operative iPTH levels were 2113 ± 787.2 and 106.2 ± 84.62 pg/ml, respectively (*p* < 0.05). The pre- and post-operative serum calcium levels were 2.39 ± 0.22 and 1.77 ± 0.30 mmol/l, respectively (*p* < 0.05). The pre- and post-operative serum inorganic phosphorus levels were 2.10 ± 0.47 and 1.43 ± 0.40 mmol/l, respectively (*p* < 0.05). The 6- and 12-month post-operative iPTH levels were 111.1 ± 56.57 and 117.6 ± 97.08 pg/ml, respectively; the 6- and 12-month post-operative iPTH level comparisons were not significantly different (Table 1).

**Table 1.** Laboratory data of patients with nonenhancement in CEUS before or after RFA.

Time	PTH (pg/ml)	Ca (mmol/l)	P (mmol/l)
preoperative	2113 ± 787.2	2.39 ± 0.22	2.10 ± 0.47
immediately	292 ± 121.8	2.24 ± 0.18	1.77 ± 0.31
1-day	106.2 ± 84.62	1.77 ± 0.30	1.43 ± 0.40
2-day	95.16 ± 100.7	1.70 ± 0.46	1.08 ± 0.35
6-month	111.1 ± 56.57	2.24 ± 0.28	1.49 ± 0.51
12-month	117.6 ± 97.08	2.36 ± 0.36	1.61 ± 0.45

## Discussion

Along with the development of disease and maintenance, a longer duration of dialysis in uremic patients can lead to bone mineral metabolic abnormalities, including calcium, phosphorus, iPTH, and vitamin D, bone turnover and mineralization, and complications, such as blood vessel or other soft tissue calcifications due to SHPT and renal bone disease, which have a severe impact on the quality of life and survival [1]. Intact PTH has a short half-life (approximately 2 min) in patients with normal renal function and approximately 5 min in patients with chronic renal failure [14]. The iPTH results from postoperative day 2 can be used as an important criterion for a successful PTX. Lokey concluded that an iPTH level <300 pg/mL on post-operative day 1 was consistent with successful RFA [15]. Indeed, an iPTH level <300 pg/mL is the indicator that most surgeons utilize [15]. Patients with refractory SHPT require surgery; however, surgery is challenging because the incision is too large, the resection is not sufficient to achieve a curative effect, excessive resection leads to parathyroid reduction, and the anesthesia risk is too high for patients with heart and/or lung disease. Because RFA is minimally invasive, more and more clinicians have attempted to treat SHPT with RFA in clinical studies and have concluded that ablation is a good treatment for SHPT [8,16,17]. We ablated the parathyroid gland under the guidance of CEUS, the results of which were similar to previous studies. RFA of SHPT is very difficult relative to other organs because the ablation level determines efficacy. CEUS is the key to treatment. CEUS not only guides the operation, but also evaluates the curative effect. Thus far, the role of RFA in the treatment of SHPT has not been established, and in the current study, we focused on the role of CEUS in RFA.

Ultrasound has a high sensitivity for normally positioned parathyroid glands(up to 90%) and a higher image resolution than CT and MRI [18]. Ultrasound, in combination with 99mTc-MIBI, has been used to locate the parathyroid gland pre-operatively, but ultrasound has an important role in describing the shape and size of the parathyroid gland. CEUS has higher spatial resolution than ultrasound, especially when ultrasound cannot distinguish parathyroid nodules from the thyroid or surrounding tissues. CEUS provides an index to judge the perfusion of nodules. Like a previous study [18], the parathyroid gland exhibits rapid hyper- and iso-enhancement [15,16]. These findings may increase one’s confidence in the diagnosis of hyperparathyroidism. Most parathyroid glands are spherical or ovoid, somewhat flattened bodies [18]. The patient with an inverted comma-shaped parathyroid gland had a pre-operative omission, thus it is important to have an ultrasound cross-intersect method and an enlarged scope of scanning pre-operatively. The parathyroid gland is normally located in the "dangerous triangle" of the neck, which is adjacent to the recurrent laryngeal nerve and neck vasculature, where ablation is likely to cause irreversible damage to the surrounding tissues [19,20]. CEUS makes it easier to distinguish the shape and extent of the parathyroid gland so that we can devise an accurate and detailed surgical plan. The liquid isolation plays a key role in the protection of the recurrent laryngeal nerve and its surrounding tissues, and the establishment of a barrier significantly reduces the incidence of complications [8,21]. In the process of separating the gland from the surrounding tissues, it is difficult to avoid slight and harmless bleeding which may make the image turbid. The hyperplastic parathyroid gland is located behind the thyroid gland and is hypoechoic. When the echo of the liquid is consistent with that of the gland, it is difficult to judge the size and location of the gland by ultrasound; however, after the contrast agent was injected, the hidden glands were enhanced in contrast to the surrounding tissues. As a result, we accurately judged the relationship between the location of the parathyroid gland and the surrounding tissues, and were able to prevent complications. In our study, under CEUS guidance, we used RFA to achieve hemostasis in a patient. This finding confirmed that CEUS not only guided our ablation scheme, but also helped us identify the cause of bleeding complications and provide intervention. Accurate assessment of blood perfusion of the lesion post-operatively is crucial for treatment planning [12]. In our study, CEUS provided guidance to complete the task immediately post-operatively. RFA was terminated in four patients because of intra-operative complications. One day post-operatively we performed CEUS and noted that there was slight enhancement in three glands, which indicated that the ablation was incomplete for the following reasons. The ablation process may lead to temporary occlusion of the microvessels, resulting in a blood supply that did not accurately reflect the nodules. In addition, a previous study suggested that the intensity of the ultrasonic echo decreases with the depth of the focus [13], and the deeper the focus, the less the blood flow. In addition, after we set up the isolation zone and performed ablation, the gas produced after ablation and the tissue carbonization affected the quality of contrast-enhanced ultrasound images. On post-operative day 2, with improvement in the ablated area, the image quality was greatly improved, which better reflected the actual blood supply to the gland than immediately after ablation. RFA is minimally invasive and it is easy to perform RFA under CEUS guidance after a short time has elapsed. Together, these findings indicated that CEUS plays an important role in the entire operative process.

This study had several limitations. First, the sample size was small. Second, because the ablations were all performed by a single surgeon, the results may be biased.

RFA guided by CEUS is effective and feasible for the treatment of parathyroid hyperplasia in the case of four or more glands. CEUS provides a guiding role to the surgeon before, during, and after RFA. The results of CEUS on post-operative day 2can better reflect the effect of RFA compared with an immediate assessment. RFA of the parathyroid requires more experience and appropriate timing of ablation.
